# International survey to identify diagnostic needs to support malaria elimination: guiding the development of combination highly sensitive rapid diagnostic tests

**DOI:** 10.1186/s12936-017-2037-z

**Published:** 2017-09-22

**Authors:** Ana Campillo, Jennifer Daily, Iveth J. González

**Affiliations:** 1 The Foundation for Innovative New Diagnostics (FIND), Geneva, Switzerland; 2 FIND, Geneva, Switzerland

**Keywords:** Malaria, Diagnostics, Rapid test, Elimination, Survey, Active case detection

## Abstract

**Background:**

In malaria elimination settings, the very low levels of transmission now being attained present challenges that demand new strategies to identify and treat low-density infections in both symptomatic and asymptomatic populations. Accordingly, passive case detection activities need to be supplemented by active case detection (ACD) strategies with more sensitive diagnostic tools. Malaria rapid diagnostic tests (RDTs) have provided low- and middle-income countries with unprecedented access to malaria diagnostics. Nevertheless, conventional RDTs miss a potentially important proportion of sub-microscopic infections. Therefore, new combination highly sensitive (HS-)RDTs, able to detect low parasite densities and identify all infected individuals, could support countries implementing ACD strategies for radical cure to accelerate malaria elimination. To address this need, an on-line survey was conducted to gather information from malaria control programme representatives to guide the development of next-generation RDTs.

**Results:**

Most of respondents confirmed that ACD was a common activity in their programmes (56/75; 75%). Although microscopy was the preferred method in case management and reactive case detection, RDTs were the primary diagnostic tests used in proactive case detection (31/75; 41%). In terms of preferences for species detection in a new combination HS-RDT, data was not one-directional. Survey respondents slightly preferred the *Pf/Pv/Pan* combination (42%; 21/50), while *Pf/Pan* was more popular among end-users. Survey respondents also valued a low-cost (< $1.00 USD), lightweight and portable test, able to detect asymptomatic infections and differentiate species, as well as provide immediate results that could be interpreted with the naked eye. In addition, respondents were open to new tests and even to replace the existing ones for ACD (63%; 47/75).

**Conclusions:**

This survey provided valuable information on the use and current limitations of ACD, on the primary product characteristics for a next-generation combination HS-RDT to support ACD and radical cure, and on the potential adoption of such a test, if available, to support malaria elimination.

## Background

Malaria continues to be a significant public health concern, responsible for approximately 212 million new cases and 429,000 deaths in 2015 [1]. Nevertheless, scale-up of malaria control efforts has contributed to tremendous reductions in disease burden and, as a result, elimination is currently considered a target in numerous countries. Between 2010 and 2015, global new malaria cases and mortality rates fell by 21 and 29%, respectively [1] and during the same period, the World Health Organization (WHO) declared 17 countries and territories no longer endemic or having zero indigenous malaria cases [2]. In 2015, the World Health Assembly endorsed the ambitious goal of achieving worldwide malaria elimination in 10 countries by 2020 and 35 by 2030 [3]. There is currently a strong global commitment to malaria elimination, and malaria eradication has been embraced by the Roll Back Malaria Partnership and by WHO as a long-term reality [4].

With enough resources, elimination can be achieved with current tools in countries with reasonably good economies, infrastructure, and health systems [5]. Nevertheless, the very low levels of transmission now being attained in many countries present new challenges that demand new strategies, such as active case detection (ACD), to identify and treat infections among populations who do not seek treatment [6]. ACD is defined by WHO as the ‘detection by health workers of malaria cases at community and household levels, sometimes in population groups that are considered at high risk. ACD can consist of screening for fever followed by parasitological examination of all febrile patients or as parasitological examination of the target population without prior screening for fever’ [7].

Accordingly, different ACD strategies have been developed to find subclinical infections in populations at risk and foci of transmission that may become important as elimination is approached [8, 9]. These tactics are generally split into two broad types: reactive case detection (RACD) and proactive case detection (PACD). RACD is an active surveillance method that is triggered by passively detected cases. It involves screening households or individuals within a specified area, typically a pre-determined radius around a locally acquired case, with the goal of preventing further malaria transmission by identifying additional symptomatic or asymptomatic infections [10, 11]. PACD consists of targeted or mass screening to search for cases in the community, which may include screening to find cases that are symptomatic or asymptomatic without the trigger of passively detected cases [12, 13].

Inherent to the change in focus that occurs as countries progress from control to elimination is the need to interrupt transmission which requires identification and treatment of parasite carriers with sub-microscopic infections that are still capable of contributing to transmission [6, 14, 15]. In this sense, one of the main challenges identified by the malERA Consultative Group on Diagnoses and Diagnostics was to develop more sensitive tests capable of identifying very low parasite densities in asymptomatic individuals in field settings for mass screening and treatment [15].

The WHO currently recommends malaria diagnosis either by microscopy or rapid diagnostic test (RDTs) in patients with suspected malaria prior to treatment [1]. Conventional RDTs are adequate for diagnosing people with symptomatic malaria. They have facilitated access to malaria diagnosis outside health facilities in peripheral communities beyond the reach of microscopy and have the potential to become the test format of choice for ACD due to their portability and ease of use [12, 16]. Nonetheless, current RDTs cannot detect the low-level blood-stage malaria infections that can be detected by more sensitive but complex methods such as nucleic acid amplification techniques (NAATs) [17].

Whether more sensitive tests will make a significant impact on malaria elimination is still unclear [18], a new combination highly sensitive (HS-)RDTs, able to detect low-density parasitaemia due to any *Plasmodium* species and to identify all infected individuals, could support countries implementing strategies to accelerate the elimination of all forms of malaria. More specifically, in ACD, a combination HS-RDT could detect people infected with parasite densities below the limit of detection of current RDTs, prior to the development of symptoms and before transmission to others in their community. Compared with ACD using existing diagnostics, a combination HS-RDT could be more cost-effective and practical because programmes could detect a greater proportion of infections, spending less time in the field tracking every case and launching easier-to-implement screening and treatment campaigns (i.e. minimal infrastructure requirements, less labour-intensive).

To define how to improve next-generation RDTs, it is necessary to understand where current diagnostics are falling short, in particular in supporting ACD activities and to identify the primary customer preferences. Therefore, an on-line survey of malaria control programme representatives from endemic countries was conducted to gather information to support the development of a new combination HS-RDT for elimination, in an informed and validated way.

## Methods

Survey design, data sources and analysis methods are described below.

### Data collection

#### Survey with national malaria control programmes

An on-line, anonymous survey consisting of 20 questions was conducted between August and September 2016. The survey was accompanied by a description of survey objectives and definitions of ACD strategies. It inquired about current practices for malaria control and elimination (i.e. PCD, ACD), use of currently available diagnostic tests, and preferred characteristic for improved RDTs. The survey was available in English, French and Spanish. Invitations to participate were sent by email to 334 individuals from 104 malaria endemic countries included in FIND’s database. The target audience was national malaria programmes, as well as local institutions working closely with them on implementation of control and elimination strategies.

#### End-user survey

An email (in English and Spanish, as needed), including a brief questionnaire with 6 questions, was sent to 28 national or regional diagnostics supervisors from 14 different malaria endemic countries in November 2016. Questions focused on test type preferences and on the challenges in current malaria RDT use, in particular common operator errors.

#### WHO data

Published data collected by the WHO/Global Malaria Programme for the 2015 World Malaria Report was also used for the analysis. This dataset included information regarding prevalence of *Plasmodium vivax* and the current phase of WHO’s malaria programme in WHO countries (control, pre-elimination, elimination, prevention of re-introduction, and malaria free), and ACD policies.

### Analysis

Data analysis involved translation to English, formatting, and merging survey responses with a data set of basic statistics on the countries (WHO region, WHO programme phase, and *P. vivax* prevalence). Absolute and relative number of responses was determined for each question, providing counts and percentages, respectively. Aggregated tables, as well as pie and column charts were generated to represent results. Major differences when comparing survey data with information available from WHO (e.g. region, programme phase, and *P. vivax* prevalence), are highlighted below in “Results” and “Discussion” sections.

## Results

### Survey demographics

**Fig. 1:**
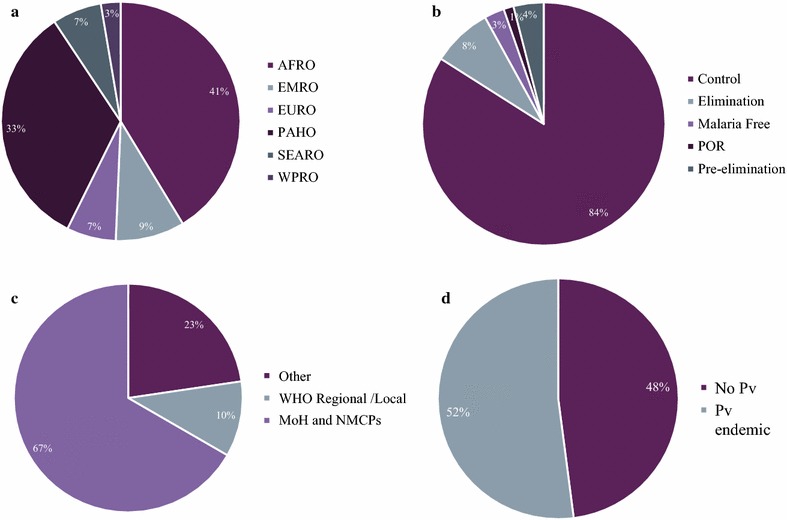
Survey demographics. **a** Respondents by region: African (AFRO), Americas (PAHO), Europe (EURO), the Eastern Mediterranean (EMRO), South-East Asia (SEARO) and the Western Pacific (WPRO). **b** Represented countries by WHO programme phase: control, pre-elimination, elimination, prevention of re-introduction (POR), and malaria free. **c** Respondents by affiliation: Ministries of Health (MoH) and National Malaria Control Programmes (NMCPs) from WHO regional offices and others (i.e. researchers, non-profit organizations). **d** Countries where *Plasmodium vivax* (*Pv*) is present or absent. All values are represented in percentages

Replies were received from 75 (22%) of the 334 contacted individuals, representing a total of 48 malaria endemic countries. Almost three quarters of the participants in the survey were based in the WHO AFRO and PAHO regions (31/75; 41% and 25/75; 33%, respectively; Fig. 1a). Most of the represented countries were those classified as being in the “Control” WHO programme phase (63/75; 84%; Fig. 1b). Questionnaire respondents were mostly from national malaria control programmes (NMCPs) and ministries of health (MoH) (50/75; 67%; Fig. 1c), as well as from countries where *P. vivax* is prevalent (38/73; 52%; Fig. 1d).

Results were also compared with World Malaria Report (WMR) data [1] to understand how representative the survey data were. EMRO (9% in the questionnaire versus 14% in the WMR) and Asian regions (2–5% vs 10–10% in the WMR; SEARO-WPRO, respectively) were underrepresented while PAHO (33% vs 19% in the WMR) was overrepresented. Countries in prevention of reintroduction and pre-elimination phases were also underrepresented in the survey (1 and 4% vs 7 and 8% in the WMR, respectively), while ‘control’ countries were highly represented (84% vs 70% in the WMR). Countries with *P. vivax* prevalence were approximately equally represented in both data sets (42% vs 47% in the WMR).

### Current diagnostics use

Inquiry about the use of malaria diagnostic tests showed that microscopy was the primary diagnostic test used (132/300; 44%), particularly in countries with *P. vivax* prevalence (80/152; 53%). Survey results indicated that microscopy was the preferred method in case management activities (49/75; 65% of all respondents), with RDTs (24/75; 32%) as a second test. Regarding the primary RDT type used, *Pf/Pan* test was the most commonly used test (28/75; 37%). Nonetheless, in countries with *P. vivax* prevalence, the *Pf/Pv* test was the most commonly used test (17/38; 45%), predominantly in the PAHO region.

Even though the majority of participating countries were classified by WHO as “control phase” countries (63/75; 84%), many respondents indicated that their programmes were performing ACD activities (56/75; 75%). Most of these countries (53/56; 95%) would perform more ACD activities if they had the capacity and resources. The biggest obstacle to performing more ACD was lack of sufficient budget (23/62; 37%), followed by lack of human resources (13/52; 25%) and transportation (12/59; 20%).

Microscopy (34/75; 45%) was slightly more common than RDTs (29/75; 39%) for RACD. In contrast, the primary diagnostic test used for PACD was RDT (31/75; 41%). Molecular testing was rare and only used in a few countries for surveys (13/75; 17%) and for ACD (2/75; 3%). Differences among regions were only observed in Africa, where RDTs were the primary diagnostic test used for RACD (18/31; 58%).

**Fig. 2:**
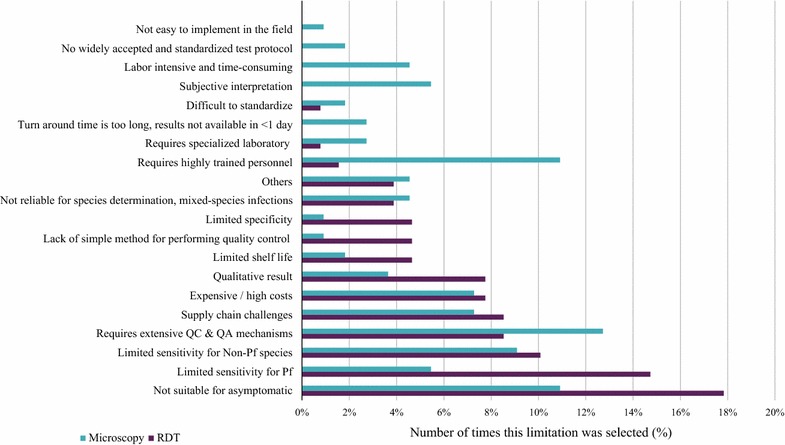
Limitations of the primary diagnostic used by program for ACD (all survey respondents). Respondents were asked to select up to three limitations. Responses were further analysed to split answers from “RDT” and “Microscopy” users. “Other” included: financial hardship, lack of confidence in suppliers, storing conditions, test acquisition, human resources, and lack of government political commitment. Columns represented the number of times that a given limitation was selected in percentage values. *QC* quality control, *QA* quality assurance

Interestingly, survey respondents were largely satisfied with their current diagnostic method for ACD (40/53; 75% were either “somewhat satisfied” or “extremely satisfied”). Nevertheless, satisfaction was slightly lower among those who rely on microscopy rather than RDTs for ACD: 30% were dissatisfied with microscopy (7/23) compared with 17% dissatisfied with RDTs (4/23). As shown in Fig. 2, the greatest limitation of malaria diagnostics for ACD was their lack of suitability for parasite detection in asymptomatic carriers, followed by their limited sensitivity for non-*Plasmodium falciparum* and *P. falciparum* species, as well as the requirement for extensive quality assurance and quality control mechanisms.

In this survey of end-users, the greatest challenge when using RDTs was to produce high quality tests that could withstand the conditions of transport, storage and use, particularly in high-temperature and high-humidity settings, with limited logistics and infrastructure. An improvement in some kit components (e.g. buffer volume, volume mark in pipettes, non-transparent cartridge color) and reagents, as well as extensive quality control and assurance mechanisms, were also seen as key factors for the success of RDT testing in the field.

### Preferred product characteristics for a new combination HS-RDT

Survey participants were asked to provide suggestions regarding their desired product characteristics for a new combination HS-RDT for ACD activities to accelerate malaria elimination (Table 1). Survey respondents were asked to assume that a new combination HS-RDT would effectively detect all species, (i.e. even those with gene deletions), would have a limit of detection ten times better than current RDTs, and would be a WHO-prequalified lateral flow assay.

**Table 1:** Key product characteristics according to survey respondents

Attribute	Preferences	Comments
Species detection combination	*Pf/Pv/Pan* *Pf/Pan*	The majority of participants preferred a *Pf/Pv/Pan* combination (42%; 21/50) while others were split between a *Pf/Pan* (36%; 18/50) and a *Pf/Pv* (18%; 9/50) test In the end-user survey, the *Pf/Pan* test was preferred (74%; 14/19)
Price	Less than $1.00 USD	Most survey respondents selected $0.50–0.99 USD as a reasonable price (52%; 35/67)
Shelf life and stability	24 month shelf life at < 40 °C	The preferred shelf life was 2 years (79%; 59/75). Among different temperature options (30, 35, 40, 45 °C) preferred requirement was 40 °C (39%; 29/75)
Sample type	Peripheral whole blood from finger-prick is acceptable	Respondents were more open to saliva sampling (74% would use it for case management, ACD or both), but were less open to urine, breath or transdermal sample types (60, 54 and 49% respectively would use it for case management, ACD or both)
Packaging	Single use kits	Single kits, instead of bulk packaging with shared buffer, was most common (71%; 53/75)
Reader	Optional	Respondents indicated that the use of a reader was a moderate priority: 49% (37/75) of participants answered that it was “very likely” that their programme would implement an RDT with a reader for ACD. Among the capabilities provided by a reader, “enhanced sensitivity” was the most valued (65%; 49/75)

The percentages represent the total number of times the particular benefit was selected compared to the total number of selections made for this question. The absolute number of responses is also provided

**Fig. 3:**
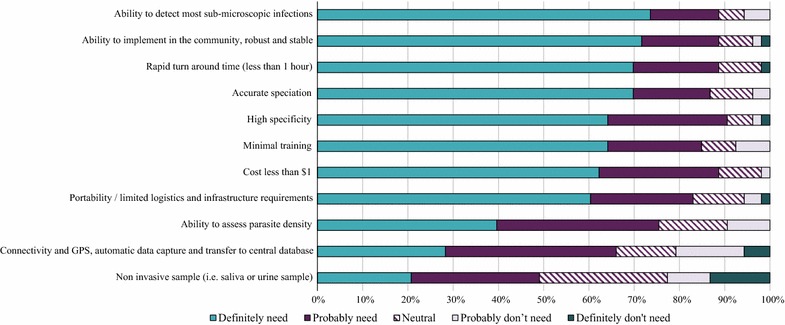
Importance of various features of a new combination HS-RDT for active case detection. Respondents were asked to select the importance of each feature in terms of “definitely need”, “probably need”, “neutral”, “probably don’t need”; and “definitely don’t need”. Columns represented the percentage of importance for each feature. *GPS* global positioning system

In terms of performance, when asked about the importance of other features for a diagnostic test for ACD, respondents underscored, as “definitely need”, the ability to detect most sub-microscopic infection (39/53; 74%), accurate speciation (37/53; 70%) and high specificity (34/53; 64%). A portable test with rapid turnaround time that can be used in the community, with minimal training needs and infrastructure level, were priorities from an operational point of view (Fig. 3).

### Potential adoption

Assuming that price was not an issue, respondents were favorable to implementing a combination HS-RDT ten times more sensitive than today’s best combination RDTs; 63% (47/75) and 29% (22/75) answered either “extremely likely” or “very likely” to implement it, respectively. From those, the following potential ACD activities where the new test would be used were indicated: PACD in high risk populations (19%; 56/293); reactive case detection (18%; 53/293); surveys with provision of treatment (15%; 45/293); PACD of high risk locations (15%; 43/293); screening pregnant women (14%; 41/293); and border screening (14%; 40/293).

Of respondents that would adopt the new highly sensitive RDT for ACD, almost half indicated they would “largely replace existing tests for ACD with combination HS-RDTs”, while the remainder pointed out they would “partially replace their existing tests with combination HS-RDTs” (46%; 32/69 in both cases). Beyond ACD and surveys, respondents would consider using the new test for travelers (27%; 50/188), while antenatal care (24%; 45/188) was also considered.

The survey also asked respondents what, in their opinion, would be the main benefits of a combination HS-RDT: ease of use/minimal user training (46/262; 18%), followed by an improved sensitivity for *P. falciparum* (44/262; 17%) and non-*P. falciparum* species (38/262; 15%) were the main benefits selected most often by participants.

## Discussion

The results of the online survey allowed us to understand the perceived limitations of existing tests for ACD, preferred key product characteristics for a new combination HS-RDT and how this test might be adopted if available.

Considering that this survey was based on voluntary participation, the response rates were satisfactory, in particular the rates achieved for the main survey target were high: NMCP/MoH represented two-thirds of respondents and are central to the decision-making process for adoption of a new product. Overall, the survey was representative of malaria endemic countries, when comparing survey responses with WMR data [1]. Only a few discrepancies in terms of regions and programme phase representation were observed.

Despite the fact that the WHO guidance is not clear on when to perform ACD or the benefits of this strategy [8, 19, 20], ACD is a common activity [21, 22] and many countries have policies in place for various forms of ACD [1]. In agreement with this, only a quarter of survey respondents were not currently implementing ACD activities. For those performing ACD, microscopy was the preferred method. Nevertheless, RDT use was dominant in the African region as shown in other studies [23, 24]. For PACD, RDTs were more common than microscopy, and again, this was driven by African respondents. Well-known examples of large-scale ACD activities using primarily microscopy has been reported in countries from other WHO regions, such as Brazil [11].

Although the survey indicated that a high number of countries are performing ACD and using RDTs for such activity, it has been reported that testing volumes overall are small and that in fact microscopy dominates ACD [25]. Nevertheless, the data highlighted that respondents using RDTs for ACD were largely satisfied with their diagnostic method, compared with those using microscopy. In addition, and in agreement with other authors [6, 14, 15], the lack of sensitivity for parasite detection in asymptomatic carriers was pointed out as the greatest limitation of current diagnostics for ACD, particularly among RDT users. In this regard, some form of ACD using a more sensitive rapid diagnostic tool would be useful, whether it be for parasite clearance or information gathering.

In terms of the preferred product characteristics for a new combination HS-RDT, survey respondents vary in their preferences for the species detection combination. Overall, the data regarding the optimal combination of test lines for a new highly sensitive multi-species RDT was not one-directional. Although there was a slight preference for a more sensitive *Pf/Pv/Pan* test, particularly in elimination countries, this preference was less dominant for countries with *P. vivax* prevalence, where a *Pf/Pv* was prioritized (e.g. PAHO), and for the AFRO and EMRO regions, where a *Pf/Pan* test was preferred. Nonetheless, a RDT with three test lines was inconsistent with consumer’s desire for low prices and with end-user feedback about difficulties of interpreting a test with multiple lines. Furthermore, technical feasibility and cost of producing this type of test would be challenging.

Because test type (i.e. the combination of species detection test bands on the RDT) is a critical component of a new combination HS-RDT, additional data analysis was conducted to better understand the options. In the limited survey of end-users, a *Pf/Pan* test was preferred. The general feeling was that a combination HS-RDT with just two test lines—including one for Pan—was easy to interpret with the proper training and able to detect all species. Bell et al. [26] also referred to a *Pf/Pan* test as the optimum requirement in *P. falciparum*-predominant areas and the minimum one in mixed *P. falciparum/non*-*P. falciparum* areas for parasite screening activities. Furthermore, *Pf/Pan* and *Pf/Pv* combination tests already exist and are widely used for case management. Nonetheless, for ACD and elimination purposes, the first option, a *Pf/Pan* test, would not allow targeted radical cure of *P. vivax* and *Plasmodium ovale* infections while the second option, a *Pf/Pv* test, would miss infections due to other *Plasmodium* species. Specific detection of *P. vivax* and *P. ovale* infections is needed in order to provide targeted radical cure and eliminate all forms of malaria. Additional analysis for product interpretation, advantages and disadvantages of different possible combination tests is presented in Table 2.

**Table 2:** Analysis of test type for a combination HS-RDT to support ACD

Test type	Interpretation and treatment	Advantages	Disadvantages	Comments
*Pan/Pf/Pv*	*Pan(*+*)/Pf(*+*)/Pv(*+*)* → ACT + PQ *Pan(*+*)/Pf(*+*)/Pv(*−*)* → ACT *Pan(*+*)/Pf(*−*)/Pv(*+*)* → CQ + PQ *Pan(*+*)/Pf(*−*)/Pv(*−*)* → CQ	Detects all species Differentiates *Pf* Allows targeted *Pv* radical cure	Does not target *Po* radical cure	Helpful for surveillance in drug resistant areas because differentiates *Pf* Difficult interpretation by end user Technically challenging
*Pan/Pf*	*Pan(*+*)/Pf(*+*)* → ACT *Pan(*+*)/Pf(*−*)* → CQ(+PQ)	Detects all species Differentiates *Pf*	*Pan(*+*)/Pf(*+*)* could be a mixed infection requiring PQ *Pan(*+*)/Pf(*−*)* would not receive PQ if *Pv* not confirmed	Helpful for surveillance in drug resistant areas because differentiates *Pf* 29% of current RDT volume market Common, familiar format
*Pf/Pv*	*Pf(*+*)/Pv(*+*)* → ACT + PQ *Pf(*+*)/Pv(*−*)* → ACT *Pf(*−*)/Pv(*+*)* → CQ + PQ	Differentiates *Pf* Allows targeted *Pv* radical cure	Does not detect *Pm/Po/Pk* Does not target *Po* radical cure	Helpful for surveillance in drug resistant areas because differentiates *Pf* 6% of current RDT volume market
*Pf/Pvom*	*Pf(*+*)/Pvom(*+*)* → ACT + PQ *Pf(*+*)/Pvom(*−*)* → ACT *Pf(*−*)/Pvom(*+*)* → CQ + PQ	Differentiates *Pf* Allows targeted *Pv* and *Po* radical cure	Over-treatment of *Pm* with PQ Does not detect *Pk*	Helpful for surveillance in drug resistant areas because differentiates *Pf* Commercial product available
*Pan/Pvo*	*Pan(*+*)/Pvo(*+*)* → ACT + PQ *Pan(*+*)/Pvo(*−*)* → ACT	Detects all species Allows targeted *Pv* and *Po* radical cure	Does not differentiate *Pf*	Shift from CQ to ACT for *non*-*falciparum* Limited surveillance data on species; speciation at reference lab required
*Pan only*	*Pan(*+*)* → ACT + PQ	Detects all species	Over-treatment of *Pf/Pm/Pk* with PQ Does not differentiate *Pf*	Shift from CQ to ACT for *non*-*falciparum* Limited surveillance data on species; speciation at reference lab required

This analysis assumes that all test lines have the same limit of detection required to detect sub-microscopic infections and that glucose-6-phosphate dehydrogenase (G6PD) deficiency testing is available and done when required

HS-RDT (high sensitive rapid diagnostic test); ACD (Active Case Detection); Pan (all Plasmodium species); Pf (*Plasmodium falciparum*); Pv (*P. vivax*); Pvom (*P. vivax, P. ovale,* and *P. malariae*); Pvo (*P. vivax* and *P. ovale*); Pk (*P. knowlesi*); ACT (artemisinin-combination therapy); PQ (primaquine); CQ (chloroquine); NAATs (nucleic acid amplification techniques)

Similar to other studies and work on the optimal diagnostic tests for malaria elimination [15, 25–27], survey respondents also valued a low-cost (< $1.00 USD), lightweight and portable test, with the ability to detect asymptomatic infections and different species, as well as provide immediate results that could be interpreted with the naked eye (although a reader would be acceptable). Long-term stability and resistance to high temperatures, as well as packaging of tests into individual kits, were also important to survey respondents.

Despite a relatively high satisfaction rate with existing tests for ACD, respondents were favorable to new tests and even to replacing existing ones. Particularly, MoH and NMCPs considered many potential ACD activities where they would use the new test, including for PACD in high-risk populations, RACD, and in surveys. In an ideal scenario, a highly sensitive field diagnostic test would increase effectiveness of targeted or mass screen-and-treat campaigns to near effectiveness of adequate mass drug administration campaigns while avoiding overtreatment of uninfected individuals [15, 28].

Recently, and after completing the survey, a new HRP2-specific highly sensitive RDT was launched into the market (Alere™ *malaria Ag Pf ultrasensitive,* catalog number #05FK140). While research data on the potential role and impact of this new *P. falciparum*-specific HS-RDT for ACD is still not available, this test is in line with most of the requirements highlighted by survey respondents and meets some of the key product characteristics, such as low-cost (USD$0.99), packaging (single use kit) and type of samples used (finger-prick blood, 5 µL).

### Study limitations

It is important to note that this study had limitations and is thus not the sole consideration in developing product attributes for a new diagnostic test. The sample size for the survey was limited and probably those who were interested in RDTs were more likely to participate. Some regions and endemicity levels were over-represented and for some questions results were not unidirectional. In addition, perspectives from end-users were certainly biased toward case management, given the challenges of identifying and communicating remotely with end-users who regularly conduct ACD. Finally, the occurrence of mistakes in the survey analysis cannot be discounted, although large efforts were invested in accurate translation and detailed cross-checks to solve any detected discrepancies or omissions.

## Conclusions

The ideal diagnostic test for malaria elimination does not exist yet, but this does not stop countries from implementing malaria elimination strategies, such as ACD with the tools available today. An effective screening tool to support accelerating elimination needs to provide a result rapidly enough to enable prompt treatment of positive cases and to distinguish between species to guide treatment decisions. In this regard, a next generation combination HS-RDT able to detect sub-microscopic malaria infections (symptomatic or asymptomatic), as well as to differentiate species requiring radical cure (*P. vivax* and *P. ovale*), could contribute to meeting the ambitious timelines for malaria elimination.

## Abbreviations

ACD: active case detection
AFRO: WHO Regional Office for Africa
EURO: WHO Regional Office for Europe
EMRO: WHO Regional Office for the Eastern Mediterranean
FIND: Foundation for Innovative New Diagnostics
HS: highly sensitive
MoH: Ministry of Health
NAATs: nucleic acid amplification techniques
NMCP: National Malaria Control Programme
PACD: proactive case detection
PAHO: Pan American Health Organization
*Pan*: *all Plasmodium species*
*Pf*: *Plasmodium falciparum*
POR: prevention of re-introduction
*Pv*: *Plasmodium vivax*
RDT: rapid diagnostic test
RACD: reactive case detection
SEARO: WHO Regional Office for South-East Asia
USD: United States Dollar
WHO: World Health Organization
WMR: World Malaria Report
WPRO: WHO Regional Office for the Western Pacific
